# Dissociation, Childhood Trauma, and Emotion Processing in Individuals with Elevated Psychosis-like Experiences: A Theoretical Framework of Cognitive Integration †

**DOI:** 10.3390/brainsci16090936

**Published:** 2026-08-31

**Authors:** Caitlin Donnelly, Veronica B. Perez

**Affiliations:** Alliant University, Daley Hall 114, 10455 Pomerado Road, San Diego, CA 92103, USA

**Keywords:** psychosis spectrum risk, social cognition, emotion processing, dissociation, cognitive integration, childhood trauma, severe mental illness, psychosocial functioning

## Abstract

**Highlights:**

**What are the main findings?**

- Individuals who may be at elevated risk for psychosis spectrum illnesses demonstrate greater childhood trauma and dissociation, poorer social cognition, and lower psychosocial functioning than healthy controls.
- Dissociation moderates the relationship between psychometrically defined elevated psychosis-like symptoms and emotion processing, suggesting that disrupted cognitive integration amplifies social cognitive impairment.

**What are the implications of the main findings?**

- Disrupted cognitive integration represents a clinically meaningful vulnerability factor linking trauma-related processes with emotion processing deficits in individuals with distressing psychosis-like experiences.
- Trauma-informed interventions targeting emotion processing and social cognition may improve functional outcomes and strengthen early intervention strategies for individuals who may be at elevated risk for psychosis.

**Abstract:**

**Background**: Childhood trauma and dissociation have each been associated with psychosis-spectrum symptoms, yet their combined relationships with social cognition and psychosocial functioning remain poorly understood. Guided by a theoretical framework of cognitive integration, the present study examined whether trauma and dissociation are associated with psychosis-like symptom severity, social cognition, and psychosocial functioning, and whether dissociation moderates the relationship between psychosis-like symptoms and emotion processing. **Methods**: A community sample of 128 adults (65 individuals with psychometrically defined elevated psychosis-like experiences; 63 healthy comparison participants) completed measures of childhood trauma, dissociation, psychosis-like experiences, social cognition (emotion processing and theory of mind), and psychosocial functioning. The study employed two complementary analytic approaches: (1) categorical group comparisons to characterize clinically meaningful group differences, and (2) dimensional analyses across the full analytic sample examining associations between trauma, dissociation, and social cognition with psychosis-like symptoms and functioning, including a moderation analysis evaluating whether dissociation altered the relationship between psychosis-like symptoms and emotion processing. **Results**: Compared with healthy comparison participants, individuals with elevated psychosis-like experiences reported significantly greater childhood trauma and dissociation, poorer emotion processing and theory of mind, and lower psychosocial functioning. Across the full analytic sample, childhood trauma and dissociation were independently associated with greater psychosis-like symptom severity and poorer psychosocial functioning. Dissociation, but not childhood trauma, was associated with poorer emotion processing. Moderation analyses further demonstrated that dissociation strengthened the relationship between psychosis-like symptom severity and emotion processing, such that greater symptom severity was associated with poorer emotion processing only at higher levels of dissociation. This moderation effect was specific to emotion processing and was not observed for theory of mind after correction for multiple comparisons. **Conclusions**: These findings suggest that dissociative experiences represent an important factor associated with poorer emotion processing and psychosocial functioning among individuals reporting elevated psychosis-like experiences. Dissociation is interpreted within a theoretical framework in which disruptions in cognitive integration may contribute to social cognitive impairment. The findings support trauma-informed assessment and intervention strategies targeting emotion processing and dissociation as potential avenues for improving functional outcomes in individuals with elevated psychosis-like experiences.

## 1. Introduction

Schizophrenia is a psychotic disorder characterized by fluctuating positive symptoms (e.g., delusions, hallucinations, disorganization), negative symptoms, and hallmark cognitive impairments [1]. While symptom severity may vary, cognitive deficits remain relatively stable and are central drivers of long-term disability and poor psychosocial outcomes [2,3]. These deficits often persist despite treatment and continue to interfere with daily functioning, such that identifying early predictors of psychosocial recovery is critical for improving long-term outcomes [4].

Cognitive dysfunction is a core and enduring feature of severe mental illness (SMI), with deficits predicting functional outcomes beyond positive symptom severity [2,5]. Impairments in attention, memory, and executive functioning are well established [6,7], and social cognition has emerged as a particularly robust predictor of real-world functioning [4,8]. Theory of mind (ToM) and emotion processing (EP), in particular, are consistently linked to interpersonal functioning, community integration, and vocational outcomes across the schizophrenia spectrum [4,9,10], and emerging evidence suggests that similar associations are detectable earlier along the psychosis continuum among individuals reporting elevated psychosis-like experiences [11]. Effective emotion processing and theory of mind require integration of perceptual input, contextual information, and mental state representations [9,12]. As such, disruptions in attention, memory integration, and self-referential processing, commonly observed in dissociative symptomatology, may interfere with these abilities [13,14].

Environmental adversity is a putative candidate mechanism contributing to cognitive vulnerability. Trauma exposure is markedly elevated in individuals with psychosis-spectrum symptoms and is associated with increased symptom severity, poorer social functioning, and worse illness trajectories [15,16,17]. Beyond its psychological impact, trauma represents a developmental and neurobiological stressor capable of altering stress-response systems, frontolimbic circuitry, and cognitive control networks [18,19]. Such alterations may affect how individuals integrate internal states with external information, a process fundamental to neurocognition, social cognition, and adaptive functioning [20,21]. One underexplored factor linking trauma exposure to cognitive vulnerability is dissociation, a transdiagnostic process frequently observed following adversity [22,23]. Although dissociation is typically defined phenomenologically, and is often described as a clinical symptom, dissociation may involve fragmentation across attentional, mnemonic, emotional, perceptual, and self-referential systems, resulting in impaired integration of contextual information [20,24,25]. From this perspective, dissociation may reflect alterations in the integration of cognitive and perceptual experiences. In psychosis-spectrum populations, self-reported dissociative symptoms are elevated and associated with more severe psychosis symptoms and poorer functional outcomes [26,27,28]. Dissociative disturbances have been proposed to interfere with the integration of internal experiences with external information, potentially influencing higher-order cognition. Individuals with elevated dissociation often have difficulty recognizing emotions and inferring others’ intentions, processes that are central to effective social functioning [29,30,31,32]. Additionally, trauma-related dissociation can interfere with emotional regulation, often leading to maladaptive coping strategies like avoidance and suppression [33,34]. The present study assesses dissociation directly, thereby conceptualizing cognitive integration as a theoretical framework.

Although trauma, dissociation, and social cognitive deficits have each been independently linked to individuals experiencing distressing psychosis-like symptoms [35,36,37] and functional outcomes [36,38,39], few studies have examined their combined effects, and findings remain mixed. For instance, some emerging developmental models suggest that early relational trauma may disrupt mentalization capacities, including ToM [40,41,42], contributing to both dissociative phenomena and psychosis-related social dysfunction. While some models propose that trauma contributes to functional impairment indirectly through cognitive and social cognitive pathways [43], it remains unclear whether dissociation serves as a moderating mechanism or alters the strength of associations among symptoms and cognitive performance relationships [44,45].

As such, the aims of the current study are twofold: first, to replicate the categorical group comparisons of individuals who experience distressing psychosis-like symptoms relative to a healthy comparison group in the clinical and cognitive characteristics supported by previous work, and second, to examine the relationships across these factors. Although psychosis-like symptoms are increasingly conceptualized dimensionally, clinically meaningful group comparisons remain an important approach for characterizing cognitive and functional differences associated with elevated symptom burden. If dissociation contributes to social cognitive dysfunction, trauma-informed approaches targeting self-integration may enhance strategies for cognitive remediation [19,31]. Conversely, strengthening ToM and emotion processing may mitigate functional impairment in individuals with trauma histories and elevated dissociation. If dissociation influences the relationship between psychosis-spectrum symptoms and social cognition, it may function as a vulnerability amplifier, increasing the impact of symptom severity on cognitive processes essential for social functioning [46,47]. Accordingly, the present study examined dissociation as a framework of disrupted cognitive integration to determine whether these experiences predict psychosis-spectrum symptoms and social and role functioning, and if dissociation increases the impact of symptom severity on cognitive processes essential for social functioning. Consistent with prior literature, we hypothesized that individuals psychometrically defined with elevated psychosis-like experiences who may be at-risk for psychosis spectrum illnesses (ARp) would show higher levels of dissociation and trauma, poorer social cognition, and worse psychosocial functioning relative to a healthy comparison group (HC). We further hypothesized that trauma and dissociation would demonstrate independent associations with symptom severity and poorer functioning, with dissociation moderating the relationship between symptom severity and social cognition.

## 2. Method

### 2.1. Transparency and Openness

We report how we determined our sample size, all data exclusions, all manipulations, and all measures in the study. Data were exported into the software program Statistical Package for Social Sciences (SPSS 25.0) for further analysis. Data are available upon request. The current study design and its analysis were not pre-registered.

### 2.2. Participants

**Figure 1:**
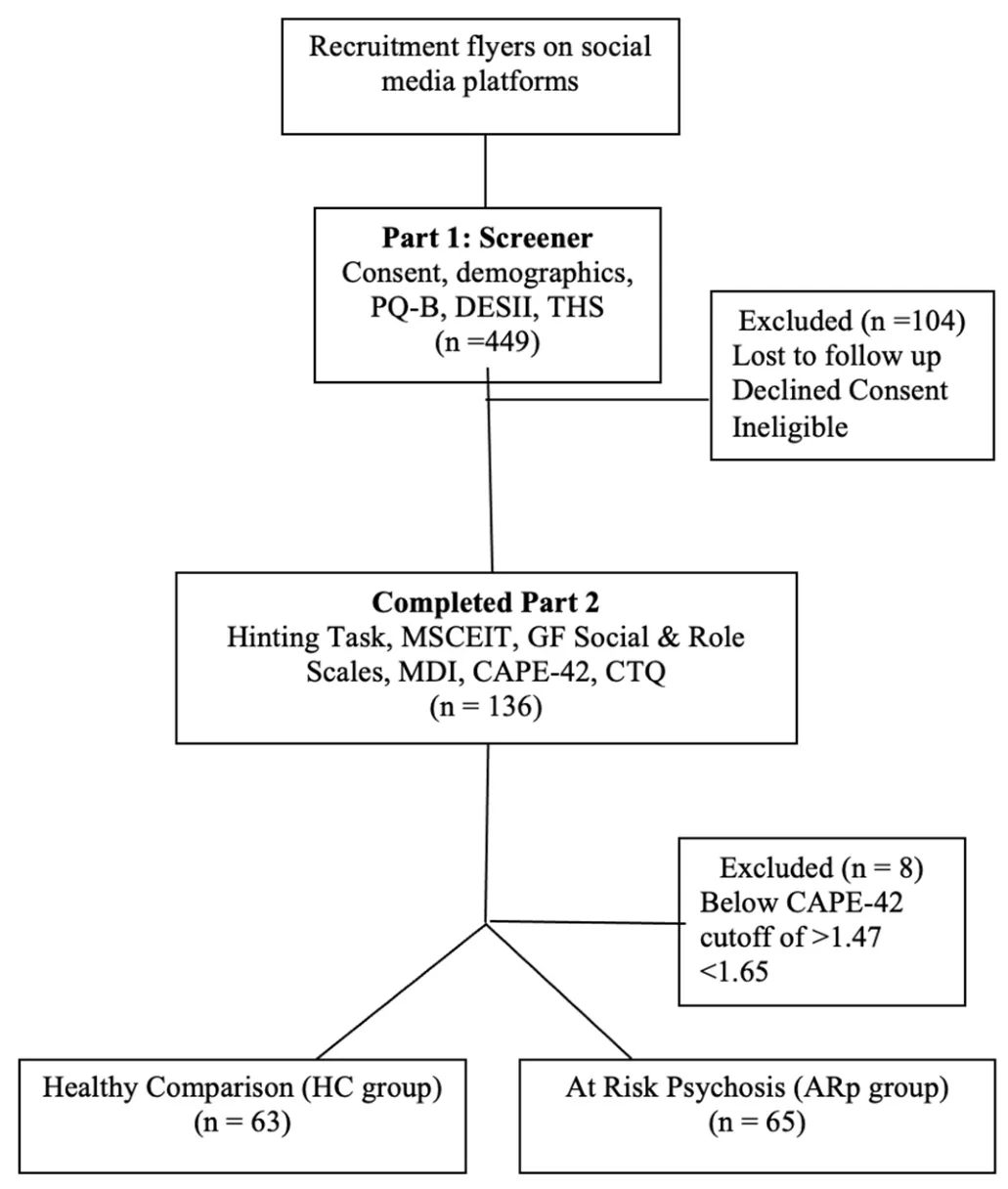
Participant Flow Diagram. Note. PQ-B = Prodromal Questionnaire Brief; DESII= Dissociative Experiences Scale; THS = Trauma History Screen; MSCEIT = Mayer–Salovey–Caruso Emotional Intelligence Test; GF Social = Social Functioning Scale; GF Role = Role Functioning Scale; CAPE-42 = Community Assessment of Psychic Experiences; MDI = Multidimensional Inventory of Dissociation; CTQ = Childhood Trauma Questionnaire Short Form.

Individuals 18–35 years old were recruited with flyers disseminated across social media platforms and through word-of-mouth. An a priori G*power 3.1 analysis indicated a minimum of 62 participants per group (ARp, HC), with an alpha of 0.05 and a power of 0.8, indicating that a total sample size of 124 participants was necessary to detect a significant change based on Cohen’s *d* = 0.80 [48], which was met. Participants completed the Prodromal Questionnaire-Brief (PQ-B) to screen for risk for psychosis spectrum symptoms and distress [49]. The total symptom score is the sum of ‘yes’ responses, ranging from 0 to 21, while the distress score is the sum of distress ratings, ranging from 0 to 105. Previous work has emphasized the importance of considering distress, impairment, persistence, and symptom context rather than item endorsement alone [50,51]. Starkowska et al. [52] suggested that clustered approaches to PQ-B classification may improve identification of clinically meaningful profiles, and Raver et al. [53] applied a composite score that emphasized distress associated with endorsement of psychosis-like symptoms. Group assignment was determined using the Psychosis Spectrum (PS) Composite score [53], computed with the formula: PS composite = (PQB Total + [PQB Distress * 3])/Total. Composite scores range from 0 to 16. The composite score creates a weighted average according to distress from the number of endorsed psychosis spectrum symptoms, using the cluster approach adapted from Starkowska et al. [52]. Participants were classified a priori using psychometrically defined criteria to compare clinically meaningful groups, consistent with the study’s first aim [54,55]. Group designs remain widely used in psychosis risk research because they permit characterization of clinically meaningful differences between individuals with elevated psychosis-like experiences and healthy comparison participants [56,57,58] before examining dimensional relationships with the analytic sample [54]. Inclusion criteria further required participants to reside in the United States, be fluent in English, and have access to a network-connected camera. A total of 128 subjects comprised two groups: risk for psychosis spectrum (ARp = 65) based on a composite score cutoff > 11 and healthy control comparison (HC = 62) based on a composite score cutoff < 3. To address floor effects among participants scoring 0 on the PQ-B, additional validation was conducted using the Community Assessment of Psychic Experience [59]. Individuals with a total score of zero on the PQ-B were retained only if CAPE-42 scores fell within established non-clinical cutoffs. Here, group assignment should be interpreted as psychometric classification rather than diagnostic confirmation of schizophrenia, first-episode psychosis, or clinical high-risk status as semi-structured interviews were not employed (see Figure 1). Exclusion criteria included substance abuse treatment within the past three months or a history of intellectual or developmental disability. Written informed consent was obtained from all participants involved in the study. This study received approval from the Institutional Review Board (IRB) of the California School of Professional Psychology (CSPP) at Alliant University in San Diego (IRB #: IRB-AY2022-2023-20 on 13 April 2023).

### 2.3. Measures

#### 2.3.1. Clinical Assessment

Psychosis spectrum symptoms were assessed using the **Prodromal Questionnaire-Brief** (PQ-B), a reliable and valid screening tool for psychosis risk (α = 0.92; 89% sensitivity) [49,60], which captures both symptom presence and related distress and effectively identifies individuals at elevated risk for psychosis. To support sample validity, PQ-B scores were corroborated from screening to testing (i.e., within 4 weeks) using the **Community Assessment of Psychic Experiences-42** [61], a well-validated measure with strong internal consistency [62]. The present sample demonstrated high correlations between the two assessments (PQ-B Total & CAPE Total: r = 0.66, *p* < 0.001; PQ-B Distress & CAPE Distress: r = 0.72, *p* < 0.001), supporting classification validity. The PS Composite was used statistically a priori as the outcome variable in regression analyses, and as the primary symptom predictor in moderation analyses.

Dissociation, conceptualized as a theoretical framework for integration of cognitive processing, was assessed using two measures. The **Dissociative Experiences Scale II** (DES-II [63]), a 28-item self-report scale assessing the frequency of dissociative experiences, demonstrates good internal consistency (α = 0.84–0.87), strong temporal stability (r = 0.84–0.92) [63], and predictive validity [64]. The **Multiscale Dissociation Inventory** (MDI [65]), a 30-item instrument assessing multiple domains of integrative disruption, including depersonalization, derealization, emotional constriction, and identity disturbance, demonstrates strong reliability and validity, with subscale alphas ranging from 0.74 to 0.94 [65,66]. DES-II and MDI statistically were used a priori as antecedents and moderator.

Lifetime trauma was assessed using the **Trauma History Screen** [67], a valid measure (r = 0.73) in which participants endorsed exposure to 14 potentially traumatic events and rated the associated distress; total trauma exposure was defined as the number of endorsed events. Childhood trauma was assessed using the **Childhood Trauma Questionnaire-Short Form** [68], a 28-item self-report measure with strong internal consistency (r = 0.66–0.92) and validity in non-clinical populations [69], yielding a total severity score. THS and CTQ-SF were used statistically a priori as antecedents.

#### 2.3.2. Assessment of Social Cognition and Function

Emotion processing was assessed using the **Mayer–Salovey–Caruso Emotional Intelligence Test** [70], a 141-item measure of emotional intelligence evaluating four branches: perceiving, using, understanding, and managing emotions [71]. The MSCEIT demonstrates strong validity and reliability, particularly in schizophrenia, with overall reliability above 0.90 and branch reliabilities ranging from 0.76 to 0.91 [12,70]. Doctoral-level research assistants were trained according to reliability protocols on the MSCEIT according to previous P.I. training in social cognition measures (personal communication, Caruso, 2022). Theory of mind was assessed using the **Hinting Task** [72], a 10-item measure in which participants infer indirect intentions from brief social interactions, with strong validity and reliability in samples who may be at-risk for psychosis [73,74]. Scores range from 0 to 20, with two points awarded per correct response. MSCEIT Total scores and Hinting Task performance were used statistically a priori as cognitive outcomes. Although the study was not powered to examine and correct for multiple comparisons across MSCEIT branches, significant correlations were observed between the MSCEIT Total scores with each of its branches (all *r*s > 0.7, all *p*s < 0.001 across Perceiving Emotions, Using Emotions, Understanding Emotions, and Managing Emotions branches; see Supplementary Materials Table S3).

Psychosocial functioning was assessed using the **Global Functioning-Role** [75] and **Global Functioning-Social** [76] scales to assess daily role and social functioning. Both scales demonstrate strong reliability in psychosis populations [77,78] and good construct validity, with moderate associations with Global Assessment of Functioning scores [77]. Doctoral-level research assistants were trained according to established NAPLS procedures for administration and scoring of the clinician-rated GF Social and Role scales [77].

### 2.4. Procedure and Statistical Analyses

Participants completed an online Qualtrics questionnaire including the PQ-B, DES-II, and THS. Eligible participants were then invited to a remote teleconference session, during which a trained doctoral-level research assistant administered the MSCEIT, Hinting Task, and GF Social and Role scales. The participants also completed additional self-report measures, CAPE-42, CTQ-SF, and MDI during this session.

The analytic strategy was organized around two complementary study aims. First, we examined categorical group differences between participants with elevated psychosis-like experiences and healthy comparison participants. Continuous clinical, trauma, dissociation, social cognitive, and psychosocial functioning measures were compared between groups using independent-samples tests. Distributional assumptions and homogeneity of variance were evaluated prior to analysis; Welch corrections were applied when the assumption of equal variances was violated, and nonparametric tests were used when distributional assumptions were not met. Specifically, the Mann–Whitney U test was used for Hinting Task performance. Effect sizes were calculated to characterize the magnitude of group differences, using Cohen’s *d* for parametric comparisons and *r* for the Mann–Whitney U test. Next, we examined dimensional associations across the full analytic sample (N = 128), which comprised participants selected from the upper and lower ranges of the PS Composite distribution. Childhood trauma (CTQ Total) and dissociation (DES-II Total) were entered simultaneously as independent variables in regression models examining psychosis-like symptom severity (PS Composite), psychosocial functioning (GF Social and GF Role), and social cognition (MSCEIT—emotion processing and Hinting Task—theory of mind) as outcomes. These models evaluated the unique associations of childhood trauma and dissociation with each outcome. In the moderation analyses, psychosis-like symptom severity (PS Composite) served as the primary predictor, dissociation (DES-II Total) as the moderator, and social cognition (MSCEIT and Hinting Task performance) as the outcomes. Thus, the statistical role of psychosis-like symptom severity differed across analyses: the PS Composite served as an outcome in the regression examining its associations with childhood trauma and dissociation and as the primary predictor in the moderation analyses. All analyses were cross-sectional and are therefore interpreted as associations rather than causal or temporal relationships. Because the full analytic sample consisted of participants selected from the upper and lower ranges of the PS Composite distribution, with participants in the intermediate range excluded prior to full assessment, the dimensional analyses do not represent associations across the complete underlying distribution of psychosis-like experiences. Accordingly, effect estimates from these analyses, particularly those involving the PS Composite, should be interpreted in the context of the group sampling strategy.

## 3. Results

All primary variables were screened for distributional assumptions. Where violations of normality or homogeneity of variance were detected, Welch corrections, nonparametric tests, or bootstrapping procedures were applied. Outliers, reflecting valid clinical variability, were retained. Chi-square tests assessed group differences in categorical demographic variables (gender, race, marital status, and education), with low-frequency categories combined or removed to meet assumptions, and an independent-samples *t*-test was used to examine age. No significant group differences were observed (see Table 1). To confirm that the ARp group reflected expected clinical patterns, scores were compared to established healthy control norms for primary measures (e.g., PQ-B, CAPE-42, MSCEIT, GF Social/Role). One-sample *t*-tests showed that the ARp group reported significantly higher psychosis-like symptoms, dissociation, trauma history, and mood symptoms, and significantly lower social cognition and social and role functioning than published HC norms, consistent with previous research (see Table 2).

### 3.1. Group Differences Between ARp and HC

#### 3.1.1. Psychosis-like Symptoms

As psychometrically defined, the ARp group demonstrated significantly higher psychosis-like symptoms than HC participants. On the CAPE-42 Total, ARp participants (*M* = 1.79, SD = 0.44) scored higher than HC participants (*M* = 1.49, *SD* = 0.25), Welch’s F(1, 102.16) = 21.18, *p* < 0.001, d = 0.81. Similarly, CAPE-42 Distress scores were higher in ARp (*M* = 49.51, *SD* = 26.11) than HC (M = 29.79, SD = 15.85), Welch’s F(1, 105.92) = 26.85, *p* < 0.001, *d* = 0.91. The PS Composite correlated with CAPE Total (*r* = 0.37, *p* < 0.001) and Distress (*r* = 0.41, *p* < 0.001), supporting convergent validity.

#### 3.1.2. Disruption in Cognitive Integration

ARp participants reported significantly higher dissociation than HC participants. For log DES-II, ARp (M = 3.02, SD = 0.82) scored higher than HC (M = 2.36, SD = 0.80), Welch’s F(1, 126) = 21.31, *p* < 0.001, *d* = 0.82. For log MDI, ARp (M = 4.08, SD = 0.32) scored higher than HC (M = 3.82, SD = 0.24), Welch’s F(1, 118.69) = 26.54, *p* < 0.001, *d* = 0.91.

#### 3.1.3. Trauma Exposure

Childhood trauma scores (CTQ Total) were significantly higher in ARp (*M* = 50.92, *SD* = 20.25) than HC (*M* = 36.48, *SD* = 11.69), Welch’s F(1, 103) = 24.63, *p* < 0.001, *d* = 0.87. Lifetime trauma scores (THS Total) were marginally different between groups (*p* = 0.059), where scores trended higher in ARp than HC.

#### 3.1.4. Psychosocial Functioning

ARp participants demonstrated significantly lower social and role functioning than HC. For GF Social, ARp (*M* = 6.80, *SD* = 1.51) scored lower than HC (*M* = 7.87, *SD* = 1.31), F(1, 126) = 18.32, *p* < 0.001, *d* = 0.75. For GF Role, ARp (*M* = 7.08, *SD* = 1.58) scored lower than HC (*M* = 8.24, *SD* = 1.00), Welch’s F(1, 108.22) = 24.81, *p* < 0.001, *d* = 0.87.

#### 3.1.5. Social Cognition

ARp participants performed significantly worse on both social cognitive measures. On the MSCEIT, ARp (*M* = 90.16, *SD* = 15.79) scored lower than HC (*M* = 100.39, *SD* = 9.70), Welch’s F(1, 106.86) = 19.63, *p* < 0.001, *d* = 0.78. On the Hinting Task, ARp scored significantly lower than HC, *U* = 1145.5, *Z* = −4.39, *p* < 0.001, *r* = 0.39.

### 3.2. Relationships Among Study Outcomes Across the Full Analytic Sample

#### 3.2.1. Trauma, Dissociation, and Psychosis Symptoms

Both dissociation and childhood trauma were positively associated with psychosis-like symptoms (Table 3). In multiple regression, log DES-II (β = 0.45, *p* < 0.001) and CTQ Total (β = 0.22, *p* = 0.010) were independently associated with PS Composite scores (Table 4A). The model accounted for 34% of variance, F(2, 125) = 32.46, *p* < 0.001. Thus, dissociation and childhood trauma contributed unique variance to severity of risk for psychosis-like symptoms.

#### 3.2.2. Trauma, Dissociation, and Functioning

Higher dissociation and childhood trauma were associated with poorer functioning (Table 4B). For GF Social, the model explained 17% of variance, F(2, 125) = 12.50, *p* < 0.001; CTQ was a significant predictor (β = −0.30, *p* = 0.002), whereas dissociation showed a trend (*p* = 0.058). For GF Role, the model explained 23% of variance, F(2, 125) = 18.63, *p* < 0.001; both dissociation (β = −0.22, *p* = 0.013) and CTQ (β = −0.33, *p* < 0.001) were significant predictors. Both higher levels of dissociation and trauma were significantly associated with and predictive of lower social and role functioning.

#### 3.2.3. Trauma, Dissociation, and Social Cognition

Higher levels of dissociation and childhood trauma were associated with poorer social cognition, particularly emotion processing. In analyses examining direct associations, dissociation was significantly associated with lower MSCEIT scores (*B* = −3.61, *p* = 0.050), whereas childhood trauma was not significantly associated with MSCEIT performance (*p* = 0.747). Neither dissociation nor childhood trauma was significantly associated with Hinting Task performance (*p*s > 0.70). Thus, higher levels of dissociation, but not childhood trauma, were associated with poorer emotion processing, whereas neither predictor was significantly related to ToM performance.

#### 3.2.4. Moderation Analyses

**Figure 2:**
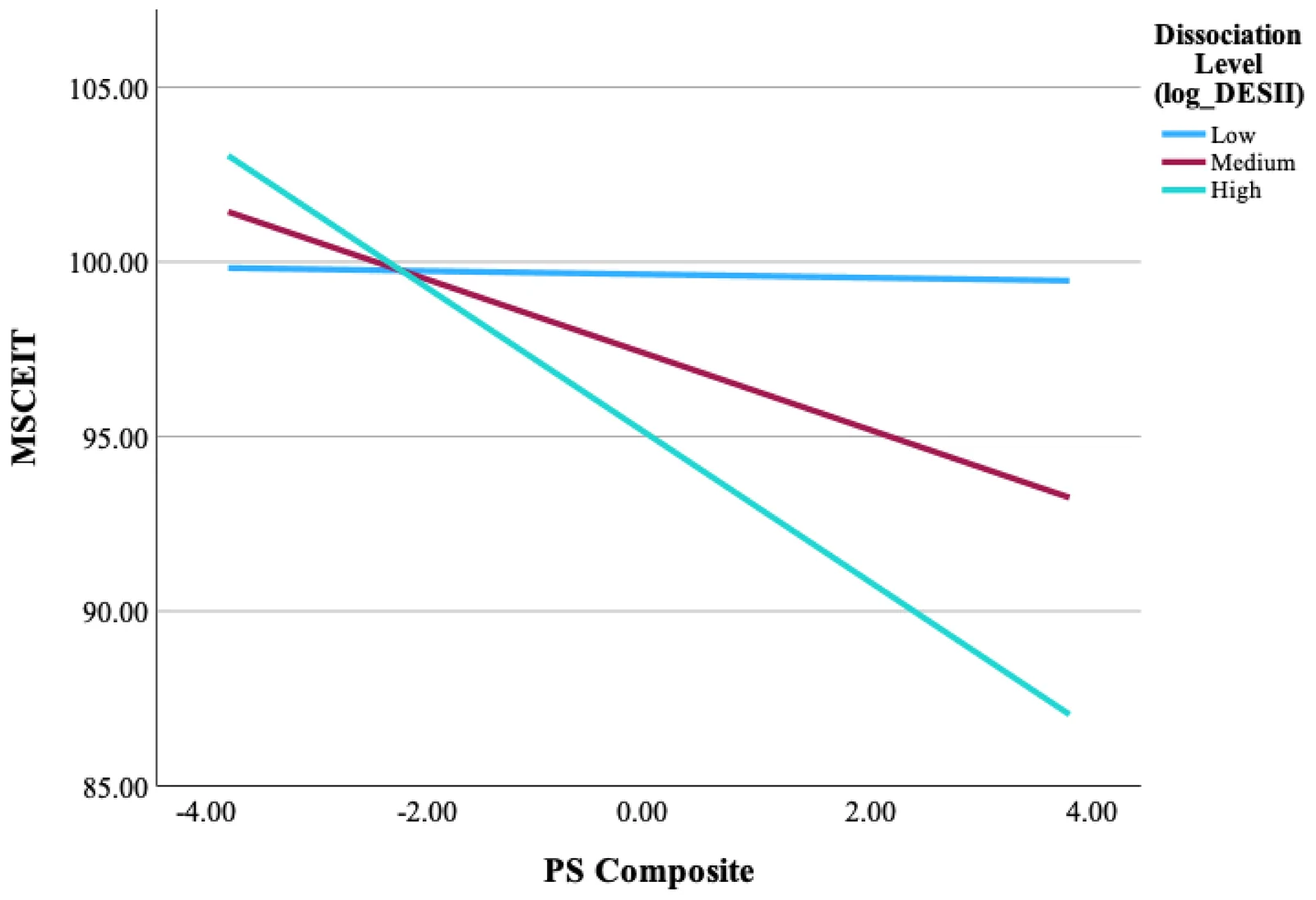
Moderation effects of dissociation on the relationship between psychosis symptom severity and social cognition—emotion processing domain. *Note.* Steeper negative slopes at high log_DESII indicate that higher dissociation strengthens the negative impact of PS Composite on MSCEIT, while the flat slope at low dissociation suggests weaker association, indicating a moderation effect. PS = Psychosis Spectrum Composite Score; MSCEIT = The Mayer–Salovey–Caruso Emotional Intelligence Test; Log_DES-II = Log-transformed Dissociative Experiences Scale; Analyses conducted with N = 128.

Our aim in examining factor relationships extended beyond identifying predictors of clinical severity and functioning outcomes to examine whether dissociation altered the relationship between psychotic-like experiences and social cognition. Accordingly, psychosis-like symptoms served as the primary predictor within this moderation model. Thus, moderation analyses examined whether dissociation altered the relationship between psychosis-like symptoms and social cognition, controlling for group. PS Composite and log DES-II were mean-centered prior to construction of the interaction terms, and HC3 robust standard errors were used to reduce sensitivity to potential heteroskedasticity. For emotion processing performance, the overall MSCEIT model was significant, F(4, 123) = 7.05, *p* < 0.001, R^2^ = 0.247. The conditional effects of PS Composite at mean dissociation (b = −1.08, SE = 0.68, *p* = 0.118, 95% CI [−2.43, 0.28]) and log DES-II at mean PS Composite (b = −2.64, SE = 1.63, *p* = 0.107, 95% CI [−5.87, 0.58]) were nonsignificant. However, the PS Composite × log DES-II interaction was significant (b = −1.18, SE = 0.41, *p* = 0.0045, 95% CI [−1.99, −0.37]), accounting for an additional 6.9% of variance in MSCEIT performance, ΔR^2^ = 0.069, F(1, 123) = 8.36, *p* = 0.0045. Conditional effects estimated at −1 SD, the mean, and +1 SD of log DES-II indicated that the association between PS Composite and MSCEIT was nonsignificant at low (b = −0.05, SE = 0.52, *p* = 0.927, 95% CI [−1.08, 0.98]) and mean (b = −1.08, SE = 0.68, *p* = 0.118, 95% CI [−2.43, 0.28]) dissociation, but was negative and significant at high dissociation (b = −2.10, SE = 0.96, *p* = 0.030, 95% CI [−4.00, −0.21]). For ToM performance, the overall Hinting Task model was also significant, F(4, 123) = 6.60, *p* < 0.001, R^2^ = 0.162. The PS Composite × log DES-II interaction was nominally significant at the conventional α = 0.05 level (b = −0.10, SE = 0.05, *p* = 0.043, 95% CI [−0.20, −0.003]) and accounted for an additional 3.1% of variance, ΔR^2^ = 0.031, F(1, 123) = 4.17, *p* = 0.043. However, none of the conditional effects of PS Composite were significant at low (b = 0.02, *p* = 0.771), mean (b = −0.07, *p* = 0.486), or high (b = −0.15, *p* = 0.227) dissociation. Moreover, because four related exploratory moderation models were evaluated using a Bonferroni-corrected threshold of α = 0.0125, the Hinting Task interaction did not meet the corrected threshold. Thus, only the PS Composite × log DES-II interaction predicting MSCEIT performance remained significant after correction (see Figure 2).

## 4. Discussion

The present study examined childhood trauma, a framework for disrupted cognitive integration (i.e., dissociation), social cognition, and functional outcomes in individuals with distressing psychosis-like symptoms. To our knowledge, this is the first study to examine how trauma and dissociation, alongside social cognitive performance, contribute to psychosis-like symptoms and functional outcomes within a sample who may be at risk for psychosis-spectrum illnesses. Prior research has established independent associations among trauma, dissociation, social cognition, and psychosis risk, but has largely considered these factors in isolation, with limited work examining their combined effects or conceptualizing dissociation as a disruption in integration that moderates these relationships. This study replicated and extended findings to individuals with elevated psychotic-like experiences to demonstrate more trauma exposure and dissociative symptoms, poorer social cognition, and lower psychosocial functioning compared to healthy controls.

The observed group differences were largely consistent with prior research in psychosis-risk samples. However, the present sample reflects elevated psychotic-like experiences rather than interview-confirmed clinical high risk (CHR) status. Nonetheless, similar to CHR studies, individuals in the current ARp group reported more childhood trauma, greater dissociative experiences, poorer functioning, and worse social cognition than healthy controls, supporting the validity of the sample and aligning with evidence that trauma exposure, social cognitive impairment, and functional decline emerge early in those who may be at risk for psychosis-spectrum conditions [58,79,80,81,82].

Interestingly, childhood trauma distinguished groups, whereas lifetime trauma did not differ significantly, contrasting prior research showing elevated lifetime trauma in at-risk populations [83,84]. This pattern suggests that developmental adversity, rather than cumulative exposure, may be more closely tied to psychosis vulnerability in this sample. One explanation may be that the THS includes a wide range of non-interpersonal events (e.g., natural disasters, accidents) that may have been less frequently endorsed in our younger sample. Some participants may also have underreported experiences not perceived as traumatic or lacking interpersonal harm. Additionally, restricted variance in trauma severity, or a possible ceiling effect in the ARp group, may have limited the ability to detect group differences.

Across the full analytic sample, both childhood trauma and dissociation contributed unique variance to psychosis-like symptoms, reinforcing their relevance to symptom expression and clinical outcomes [85]. This finding is consistent with models positioning trauma as an environmental risk factor that increases vulnerability to psychosis [86] and with work showing that altered self-experience, attentional fragmentation, and disrupted integration are associated with greater symptom severity [47]. These findings further support the view that trauma and dissociation, conceptualized as reflecting disrupted cognitive integration, are related but partially independent contributors to psychosis-spectrum expression [36,38]. Childhood trauma and dissociation also predicted poorer social and role functioning. Trauma was associated with both domains, whereas dissociation was particularly robust for role functioning, aligning with longstanding evidence that early adversity may impair psychosocial functioning [87,88], and studies linking dissociative process to long-term functional deficits [23,89]. These findings also reinforce the importance of studying functioning directly, rather than treating symptoms as the only clinically meaningful outcomes. Regarding social cognition, our findings partly aligned with expectations. ARp participants showed poorer performance than controls in both emotion processing and theory of mind, supporting previous studies that indicate social cognitive deficits can occur before the onset of full psychotic disorder [11,81,90].

Findings from the moderation analyses provided insight into potential contributing mechanisms. Higher dissociation strengthened the negative association between psychosis-like symptoms and the social cognition domain of emotion processing. At lower levels, symptoms were not significantly related to MSCEIT performance, whereas at higher levels, they were associated with poorer emotion processing, suggesting that elevated dissociation may exacerbate emotion processing difficulties as symptoms increase. This finding aligns with models proposing that dissociation amplifies risk through disruptions in attention, memory, emotion regulation, and cognitive processing [46,47]. Notably, this effect was specific to emotion processing and did not extend to theory of mind, indicating possible domain specificity. Emotion processing relies on rapid coordination of perceptual, affective, and contextual information and may be particularly vulnerable to fragmentation in attention, self-referential processing, and emotion regulation [13,20,24]. In contrast, theory of mind, as measured by the Hinting Task, may rely on more established, learned reasoning skills and may be less sensitive to subtle dissociation-related disruption. Restricted range in Hinting scores may have further limited sensitivity.

Ultimately, these findings suggest that disrupted cognitive integration, as reflected here through measures of dissociative experiences, is better understood as an associated factor rather than a vulnerability marker. Rather than explaining how trauma contributes to psychosis-spectrum symptoms or functional impairment through social cognition, disrupted integration of cognitive processes appears to strengthen the association between psychosis-like symptoms and poorer emotion processing. This distinction suggests that disrupted cognitive integration may not follow a simple linear pathway and instead alters the conditions under which domains of social cognitive dysfunction become more pronounced. In this way, dissociation functions as a clinically meaningful modifier, consistent with a theoretical framework of disrupted cognitive integration of cognitive deficit that is robustly predictive of psychosocial functioning, rather than merely a co-occurring symptom feature.

### 4.1. Limitations

Several limitations should be acknowledged. The cross-sectional design limits conclusions regarding temporal ordering, causality, and underlying mechanisms. Although dissociation moderated the association between psychosis-like symptoms and emotion processing performance, the interaction indicated that the strength of the cross-sectional association between these variables differed across levels of dissociation. Thus, it does not establish that dissociation prospectively amplifies cognitive vulnerability or contributes to subsequent psychosis-spectrum symptoms. Reverse or reciprocal relationships are also plausible. For example, difficulties in emotion processing may contribute to distress or dissociative experiences, while psychosis-spectrum symptoms and dissociation may themselves interfere with social cognitive processing. Alternatively, associations among these constructs may partly reflect shared underlying factors, such as broader psychopathology, distress, trauma-related processes, or overlapping measurement characteristics. Longitudinal studies with repeated assessments of dissociation, psychosis-spectrum symptoms, and social cognition are needed to establish temporal ordering and determine whether dissociation prospectively modifies trajectories of social cognitive functioning or psychosis-spectrum symptom expression. Participants were selected from the upper and lower ranges of the PS Composite distribution, with individuals in the intermediate range excluded. Although this approach was intentional and necessary for group comparisons, the final analytic sample does not represent the complete distribution of psychosis-like experiences. Extreme-group sampling can affect estimates of associations among continuous variables and, under some conditions, produce stronger correlations or regression coefficients than would be observed in an unselected sample [55]. Accordingly, the magnitude of the dimensional associations reported here involving the PS Composite should be interpreted cautiously and should not be considered population effect-size estimates. Replication in samples spanning the full distribution of psychosis-like experiences will be important for establishing the magnitude and generalizability of these associations. Several core variables, including trauma and dissociation, were assessed through self-report. Although the measures used in this study have strong psychometric support, they remain susceptible to recall bias, social desirability, limited insight, and recall errors [91]. Moreover, measures of psychosis and dissociation have overlapping phenomenology, which may inflate some associations and complicate construct separation [85,92,93,94,95,96]. Shared method variance is also an important consideration when multiple constructs are assessed using self-report instruments. At the same time, other outcomes of the study were performance-based, including domains of social cognition which include emotion processing (MSCEIT) and theory of mind (Hinting Task), as well as a clinician-administered semi-structured psychosocial functioning interview. Therefore, the observed relationships between self-report and performance-based measures are less likely to be attributable solely to common method bias. Formal psychometric equivalence of remote administration of the current performance-based or semi-structured functioning interviews are currently limited, though there is evidence supporting strong psychometric properties generally and all participants in the current study were assessed under the same remote conditions. Notably, the sample was recruited online, which introduces self-selection bias. The sample was predominantly White and female, which limits generalizability and may not reflect the demographic makeup of other clinical high-risk groups that have been evaluated with clinician-administered assessment tools (e.g., those from the North American Prodrome Longitudinal Study; NAPLS). While doctoral-level trained research assistants administered all performance-based or semi-structured functioning interviews blinded to group, interrater reliability was not assessed. Though remote data collection increased accessibility, it might have excluded individuals with limited internet access or digital resources, including some higher-risk populations [97,98,99,100,101,102,103]; however, smartphone access is common among individuals with psychosis-spectrum conditions, and telehealth has been successfully used to deliver mental health services to this population [104,105]. It is unclear whether participants were seeking help or engaged in treatment during the study. Furthermore, although the PS Composite has been previously described [53], additional replication in independent samples is warranted before it may be considered a broadly used psychometric classification method, as it is likely not as robust as the traditional semi-structured diagnostic interviews that have been used to classify participants into clinical high-risk or ultra-high risk for psychosis, where longitudinal conversion rates are still limited to 25–35% [58]. Finally, using multiple statistical tests raises the chance of Type I errors. Although Bonferroni correction was applied to the moderation models, the overall findings should still be interpreted cautiously.

### 4.2. Conclusions and Future Directions

The current study advances the understanding of how social cognition, trauma, and a framework for disrupted cognitive integration relate to symptoms that may present relatively early on a psychosis continuum. Findings highlight dissociative experiences as a moderating mechanism in the relationship between psychosis-like symptoms and emotion processing, suggesting that elevated dissociation may amplify social cognitive vulnerability in individuals with elevated psychotic-like experiences. ARp participants demonstrated poorer performance across domains of social cognition, underscoring its continued relevance for intervention. Dissociation, interpreted within a theoretical framework of disrupted cognitive integration, may continue to shape how symptoms relate to emotion processing and potentially identify individuals who are less resilient to increasing symptom burden. These findings support the value of examining the role of emotion processing and broader social cognitive skills within trauma-informed care. Candidate interventions such as Social Cognition and Interaction Training, metacognitive approaches, and mentalization-based therapies may be especially beneficial when integrated with approaches that address trauma-related disruption [106,107,108,109]. More broadly, this work aligns with efforts in severe mental illness research to identify modifiable correlates of cognitive dysfunction that can inform targeted, integrated interventions. Future research should examine these relationships longitudinally and in larger, more diverse samples, including help-seeking and interview-confirmed psychosis-risk populations. Expanding assessment to capture more nuanced social cognitive processes (e.g., attributional style, empathy, trust, context-sensitive mentalizing) may clarify which domains are most affected by trauma-related disruption [110,111,112]. Additionally, converging research between neurocognitive fragmentation as evidenced by deficits in attentional, mnemonic, and executive functioning processing [2,5,113,114,115] and neurobiological mechanisms, such as emotion regulation circuits, stress biomarkers, and threat detection systems [18,19,116] require further examination in individuals with distressing psychosis-like experiences. Disrupted cognitive integration reported here may shed light on how trauma and dissociation increase vulnerability for individuals with distressing psychosis-like symptoms with poor functional outcomes. Additional information about psychiatric diagnoses, medication use, current treatment, mood and anxiety symptoms, neurological conditions, sleep, and substance exposure may further characterize specific risk or treatment pathways. More precise characterization of trauma, including timing (e.g., since event, age of event, etc.), and its related symptoms (chronicity, severity, and subjective distress), may further refine our understanding of risk pathways.

Overall, trauma and disrupted cognitive integration as measured through dissociative experiences appear to play a meaningful role in symptom expression and impairment in emotional processing in individuals with elevated psychosis-like symptoms. These factors may help identify individuals who could benefit from early, targeted, trauma-informed interventions aimed at supporting functioning and reducing symptom progression.

## Figures and Tables

**Table 1:** Demographic and clinical characteristics.

	Full Sample (n = 128)		Healthy Control (HC = 63)		At-Risk Psychosis (ARp = 65)			
Variable	n	%	n	%	n	%	X^2^	*p*-Value
**Gender**	125		62		63		2.38	0.123
Male	47	38.4	28	45.7	20	32.2		
Female	77	61.6	34	54.8	43	68.6		
**Ethnicity/Race**	122						3.06	0.216
Black/African American	11	9	3	4.9	8	13.1		
White/Caucasian	97	79.5	52	85.2	45	73.8		
Other	14	11.5	6	9.8	8	13.1		
**Marital Status**	128						0.25	0.614
Single/Not Married	92	71.9	44	69.8	48	73.8		
Married	36	28.1	19	30.2	17	26.2		
**Education**	124						2.87	0.413
High School/GED	18	14.5	9	14.5	9	14.5		
Some College	41	33.1	22	35.5	19	30.6		
Bachelor’s degree	44	35.5	18	29	26	41.9		
Graduate degree	21	16.9	13	21	8	12.9		
	**M**	**SD**	**M**	**SD**	**M**	**SD**	***t*** **-Test**	***p*** **-Value**
Age (years)	27.2	4.6	27.5	4.7	26.9	4.5	0.795	0.428
**Measure**	**M**	**SD**	**M**	**SD**	**t**	**Hedge’s g**	***p*** **-Value**	
PSComposite	6.86	3.45	12.53	1.01	−12.68	2.54	<0.001	
PQB Total	4.68	4.17	8.08	4.96	−4.18	−0.74	<0.001	
PQB Distress	12.27	12.4	31.62	21.73	−6.16	1.08	<0.001	
CAPE Total	1.49	0.25	1.79	0.44	−4.56	−0.8	<0.001	
CAPEDistres	1.71	0.38	2.18	0.62	−5.15	−0.91	<0.001	
DESII	13.32	11.94	26.26	19.4	−4.53	−0.8	<0.001	
Log_DESI	2.36	0.8	3.02	0.82	−4.62	−0.81	<0.001	
MDI Total	46.03	12.4	61.26	21	−4.98	−0.88	<0.001	
DENG	12.05	3.95	15.2	4.25	−4.35	−0.77	<0.001	
DEPR	6.27	1.89	8.92	4.68	−4.81	−0.85	<0.001	
DERL	7.03	2.5	10.15	4.52	−4.19	−0.74	<0.001	
ECON	7.33	3.6	10.66	4.86	−4.4	−0.77	<0.001	
MEMD	7.86	2.96	10.2	4.2	−3.64	−0.64	<0.001	
IDDIS	5.49	1.22	6.12	2.34	−1.91	−0.34	0.057	
Log_MDI	3.82	0.24	4.08	0.32	−5.13	−0.9	<0.001	
THS Total	3.38	2.73	4.58	3.46	−2.18	−0.38	0.031	
Intp	0.95	1.14	1.62	1.49	−2.82	−0.5	0.006	
NonIntp	2.43	2.01	2.97	2.39	−1.38	−0.24	0.169	
CTQ Total	36.48	11.69	50.92	20.25	−4.92	−0.87	<0.001	
Emot Abu	8.3	3.6	12.06	5.67	−4.46	−0.79	<0.001	
Phys Abus	6.4	2.56	8.12	4.2	−2.8	−0.49	<0.001	
Sexua Abu	6.22	3.28	9.2	5.78	−3.57	−0.63	<0.001	
Emt Neglct	8.84	3.72	12.8	4.59	−5.35	−0.94	<0.001	
Phys Nglct	6.71	2.53	8.74	3.79	−3.54	−0.62	<0.001	
GF Soc	7.87	1.31	6.8	1.51	4.28	0.75	<0.001	
GF Role	8.24	1	7.08	1.58	4.95	0.87	<0.001	

*Note:* PQ-B = Prodromal Questionnaire Brief; CAPE = Community Assessment of Psychic Experiences 42 Item; DESII = Dissociative Experiences Scale; DENG = Disengagement Scale; DEPR = Depersonalization Scale; DERL = Derealization Scale; ECON = Emotional Constriction Scale; MEMD = Memory Disturbance Scale; IDDIS = Identity Dissociation Scale; THS = Trauma History Screen; CTQ = Childhood Trauma Questionnaire-Short Form; EmotAbu = Emotional Abuse scale; Phys Abu = Physical Abuse scale, SexuAbu = Sexual Abuse scale, EmotNeglct = Emotional Neglect Scale, PhyNegl = Physical Neglect Scale; MSCEIT = Mayer–Salovey–Caruso Emotional Intelligence Test, Perc Emot = Perceiving Emotions Branch, UsEmot = Using Emotions Branch, UnderstEmot = Understanding Emotions Branch, MangEmot = Managing Emotions, GF Soc = Social Functioning Scale; GF Role = Role Functioning Scale.

**Table 2:** Comparison of current study ARp scores to previously published healthy control norms.

Measure	ARp Mean	HC Norm Mean	t	*p*	Hedges’ g	Source
PQ-B Total	8.08	5.7	3.86	<0.001	0.47	Xu et al., 2016
PQ-B Distress	31.62	20.1	4.27	<0.001	0.52	
CAPE Total	1.79	1.34	8.11	<0.001	0.99	Mossaheb et al., 2012
CAPE Distress	2.18	1.7	6.21	<0.001	0.76	
DES-II	26.26	5.4	8.67	<0.001	1.06	Carlson & Putnam, 1993
DENG	15.2	12.8	4.56	<0.001	0.56	Briere, 2002
DEPR	8.92	6.9	3.49	<0.001	0.43	
DERL	10.15	8.7	2.59	0.012	0.32	
ECON	10.66	8.4	3.76	<0.001	0.46	
MEMD	10.2	8	4.22	<0.001	0.52	
IDDIS	6.12	5.8	1.12	0.269	0.14	
CTQ Total	50.92	33.7	6.86	<0.001	0.84	Bernstein & Fink, 1998
EmotAbu	12.06	7.7	6.2	<0.001	0.76	
PhysAbu	8.12	6.36	3.38	0.001	0.42	
SexuAbu	9.2	5.85	4.68	<0.001	0.58	
Emot Negl	12.8	7.9	8.6	<0.001	1.05	
PhyNegl	8.74	5.85	6.14	<0.001	0.75	
THS Total	4.58	3.2	3.22	0.002	0.4	Russo et al., 2014
Hinting	17.42	18.3	−2.54	0.013	−0.31	Corocoran et al., 1995
MSCEIT Total	90.16	106.3	−8.24	<0.001	−1.01	Frajo-Apor et al., 2016
PercEmot	97.76	103.5	−2.94	0.005	−0.36	
UsEmot	92.29	107.4	−8.23	<0.001	−1.01	
UnderstEmot	89.78	101.2	−5.66	<0.001	−0.69	
MangEmot	89.5	104.9	−11.35	<0.001	−1.39	
GF Social	6.8	8.86	−10.98	<0.001	−1.35	Carrion et al., 2019
GF Role	7.08	8.56	−7.55	<0.001	−0.93	

*Note:* PQ-B = Prodromal Questionnaire Brief; CAPE = Community Assessment of Psychic Experiences 42 Item; DESII = Dissociative Experiences Scale; DENG = Disengagement Scale; DEPR = Depersonalization Scale; DERL = Derealization Scale; ECON = Emotional Constriction Scale; MEMD = Memory Disturbance Scale; IDDIS = Identity Dissociation Scale; THS = Trauma History Screen; CTQ = Childhood Trauma Questionnaire-Short Form; EmotAbu = Emotional Abuse scale; Phys Abu = Physical Abuse scale, SexuAbu = Sexual Abuse scale, EmotNeglct = Emotional Neglect Scale, PhyNegl = Physical Neglect Scale; MSCEIT = Mayer–Salovey–Caruso Emotional Intelligence Test, PercEmot = Perceiving Emotions Branch, UsEmot = Using Emotions Branch, UnderstEmot = Understanding Emotions Branch, MangEmot = Managing Emotions, GF Soc = Social Functioning Scale; GF Role = Role Functioning Scale.

**Table 3:** Dissociation, trauma, psychosis-like symptoms, and functioning correlation matrix for the full analytic sample (N = 128).

Variable	1	2	3	4	5	6	7
1. LogDES	—						
2. Log_MDI	0.764 **	—					
3. THS Total	0.459 **	0.517 **	—				
4. CTQ Total	0.466 **	0.554 **	0.655 **	—			
5. PS Composite	0.473 **	0.491 **	0.276 **	0.490 **	—		
6. GF_Soc	−0.404 **	−0.429 **	−0.350 **	−0.370 **	−0.473 **	—	
7. GF_Role	−0.473 **	−0.492 **	−0.293 **	−0.436 **	−0.557 **	0.690 **	—

**Note.** LogDES = log-transformed Dissociative Experiences Scale; log_MDI = log-transformed Multidimensional Inventory of Dissociation Total; THS = Trauma History Screen Total; CTQ = Childhood Trauma Questionnaire–Short Form Total; PS Composite = Psychosis Symptom Composite; GF_Soc = Social Functioning Scale; GF_Role = Role Functioning Scale. All correlations are Spearman’s rho. *p* < 0.01 (2-tailed); ** *p* < 0.001.

**Table 4:** Multiple regression analyses.

*(A) Dissociation and Trauma as Correlates of Symptom Severity.*					
**Predictor**	**B**	**SE(B)**	**β**	**t**	***p***
	**PS Composite**				
Constant	2.42	0.95	--	2.55	0.01
LogDES-II	1.98	0.36	0.45	5.52	<0.001
CTQ	0.045	0.17	0.22	2.63	0.010
*(B) Dissociation and Trauma as Correlates of Social and Role Functioning.*					
**Predictor**	**B**	**SE(B)**	**β**	**t**	***p***
	**(GF Social)**				
Constant	9.24	0.46	--	21.74	21.74
LogDES-II	−0.31	0.16	-0.13	−1.91	0.058
CTQ Total	−0.03	0.01	-0.31	−3.20	0.002
	**(GF Role)**				
Constant	9.82	0.39	--	25.13	<0.001
LogDESII	−0.37	0.15	−0.22	−2.52	0.013
CTQ Total	−0.03	0.01	−0.32	−3.75	<0.001

Note. B = unstandardized regression coefficient; SE B = standard error of B; β = standardized regression coefficient. GF Social and GF Role = social and role functioning. CTQ = Childhood Trauma Questionnaire Short Form; LogDES-II = Log-Transformed Dissociative Experiences Scale-II. Analyses conducted with N = 128.

## Data Availability

We report how we determined our sample size, all data exclusions, all manipulations, and all measures in the study. Data were exported into the software program Statistical Package for Social Sciences (SPSS 25.0) for further analysis, with an average of medium to strong artifact correction for the majority of the sample. Data are available upon request. This study’s design and its analysis were not pre-registered.

## Supplementary Materials

The following supporting information can be downloaded at: https://www.mdpi.com/article/10.3390/brainsci16090936/s1, Table S1: Expanded Clinical Characteristics and Functional Outcomes to Include Distribution Metrics; Table S2: Dissociation, Trauma, Psychosis-Spectrum Symptoms, and Functioning Correlation Matrix; Table S3: Correlations Among MSCEIT Total and Branch Scores, Theory of Mind, Psychosis-Spectrum Symptoms, and Dissociation (N = 128).
