# Supplementary material for: Dissociation, Childhood Trauma, and Emotion Processing in Individuals with Elevated Psychosis-like Experiences: A Theoretical Framework of Cognitive Integration
Source: Brain Sci. 2026 Aug 31;16(9):936. doi: 10.3390/brainsci16090936 (PMC13604070; doi:10.3390/brainsci16090936)
Supplement: Supplementary file 1 [file brainsci-16-00936-s001.zip › brainsci-4437800-supplementary.pdf]

Table S1. Expanded Clinical Characteristics and Functional Outcomes to Include Distribution Metrics.

	Healthy Control n=63			*At Risk Psychosis n=65*					
**Measure**	**M (SD)**	**Skew**	**Kurt**	**M (SD)**	**Skew**	**Kurt**	**t**	**Hedge's g**	**p-value**
PSComposite	6.86 (3.45)	-0.90	-0.26	12.53 (1.01)	0.679	0.67	-12.68	2.54	<.001
PQB Total	4.68 (4.17)	0.88	0.02	8.08 (4.96)	0.61	-0.07	-4.18	-0.74	<.001
PQB Distress	12.27 (12.40)	1.07	0.40	31.62 (21.73)	1.18	1.46	-6.16	1.08	<.001
CAPE Total	1.49 (.25)	0.67	0.78	1.79 (.44)	0.75	-0.09	-4.56	-0.8	<.001
CAPEDistres	1.71 (0.38)	0.53	0.12	2.18 (0.62)	0.77	0.34	-5.15	-0.91	<.001
DESII	13.32 (11.94)	1.87	4.37	26.26 (19.40)	0.99	0.47	-4.53	-0.80	<.001
Log_DESII	2.36 (.80)	0.10	.07	3.02 (.82)	-.47	-.48	-4.62	-0.81	<.001
MDI Total	46.03 (12.40)	1.21	1.03	61.26 (21.00)	0.97	0.62	-4.98	-0.88	<.001
DENG	12.05 (3.95)	0.58	0.05	15.20 (4.25)	0.04	-0.79	-4.35	-0.77	<.001
DEPR	6.27 (1.89)	1.68	2.34	8.92 (4.68)	1.39	1.33	-4.81	-0.85	<.001

DERL	7.03 (2.50)	1.37	1.63	10.15 (4.52)	0.81	0.22	-4.19	-0.74	<.001
ECON	7.33 (3.60)	2.43	6.79	10.66 (4.86)	0.68	-0.37	-4.40	-0.77	<.001
MEMD	7.86 (2.96)	0.98	0.05	10.20 (4.20)	0.83	0.09	-3.64	-0.64	<.001
IDDIS	5.49 (1.22)	2.35	4.72	6.12 (2.34)	2.06	3.19	-1.91	-0.34	0.057
Log_MDI	3.82 (.24)	0.72	-0.14	4.08 (.32)	0.24	-0.42	-5.13	-0.90	<.001
THS Total	3.38 (2.73)	0.58	-0.63	4.58 (3.46)	0.80	0.08	-2.18	-0.38	0.031
CTQ Total	36.48 (11.69	1.54	2.50	50.92 (20.25)	0.95	0.98	-4.92	-0.87	<.001
Emot Abu	8.30 (3.60)	1.11	0.12	12.06 (5.67)	0.54	-0.76	-4.46	-0.79	<.001
Phys Abus	6.40 (2.56)	2.45	6.78	8.12 (4.20)	1.625	3.02	-2.80	-0.49	<.001
Sexua Abu	6.22 (3.28)	3.45	12.75	9.20 (5.78)	1.09	-0.07	-3.57	-0.63	<.001
Emt Neglct	8.84 (3.72)	1.02	0.17	12.80 (4.59)	0.16	-0.59	-5.35	-0.94	<.001
Phys Nglct	6.71 (2.53)	2.03	4.69	8.74 (3.79)	1.32	2.12	-3.54	-0.62	<.001
GF Soc	7.87 (1.31)	-1.43	3.14	6.80 (1.51)	-1.25	2.77	4.28	0.75	<.001
GF Role	8.24 (1.00)	-1.21	4.31	7.08 (1.58)	-1.16	2.08	4.95	0.87	<.001

Note: PS= Psychosis Spectrum Composite Score, PQ-B= Prodromal Questionnaire Brief; CAPE = Community Assessment of Psychic Experiences 42 Item; DESII= Dissociative Experiences Scale; MDI= Multidimensional Inventory of Dissociation; DENG= Disengagement Scale; DEPR = Depersonalization Scale; DERL= Derealization

Scale; ECON= Emotional Constriction Scale; MEMD= Memory Disturbance Scale; IDDIS= Identity Dissociation Scale; THS= Trauma History Screen; THS Intp = Interpersonal Trauma; THS NonIntp = Non-Interpersonal Trauma; CTQ= Childhood Trauma Questionnaire- Short Form; GAD-7= Generalized Anxiety Disorder 7 Item scale; PHQ-9= Patient Health Questionnaire; GF Soc= Social Functioning Scale; GF Role= Role Functioning Scale

Table S2. Dissociation, Trauma, Psychosis-Spectrum Symptoms, and Functioning Correlation Matrix

A) *Dissociation and Trauma Measures Correlation Matrix for HC Group = 63.*

Variable	1	2	3	4	5	6	7
1. LogDES	—						
2. LogMDI	.67**	—					
3. THS Total	.45**	.57**	—				
4. CTQ Total	.38**	.51**	.64**	—			
5. PS Composite	.53**	.54**	.39**	.33**	—		
6. GF_Soc	–.23	–.36**	–.45**	–.30*	–.41**	—	
7. GF_Role	–.37**	–.46**	–.39**	–.33**	–.58**	.66**	—

B) Dissociation and Trauma Measures Correlation Matrix for ARp Group = 65

Variable	1	2	3	4	5	6	7
1. LogDES	—						
2. log_MDI	.74**	—					
3. THS Total	.47**	.47**	—				
4. CTQ Total	.41**	.51**	.64**	—			
5. PS Composite	.17	.12	.16	.31*	—		
6. GF_Soc	–.30*	–.20	–.12	–.15	–.16	—	
7. GF_Role	–.39**	–.28*	–.12	–.34**	–.25*	.63**	—

Note. LogDESII = Log Transformed Dissociative Experiences Scale; LogMDI = Log Transformed Multidimensional Inventory of Dissociation Total; THS = Trauma History Screen Total; CTQ = Childhood Trauma Questionnaire- Short Form Total; GF Soc= Social Functioning Scale; GF Role= Role Functioning Scale. All correlations are Spearman's rho. * $p < 0.05$. ** $p < 0.01$ (2-tailed).

Table S3. Correlations Among MSCEIT Total and Branch Scores, Theory of Mind, Psychosis-Spectrum Symptoms, and Dissociation (N = 128)

Variable	1	2	3	4	5	6	7	8	9
1. MSCEIT Total	—								
2. Perceiving Emotions	.770**	—							

3. Using Emotions	.723**	.504**	—						
4. Understanding Emotions	.740**	.420**	.355**	—					
5. Managing Emotions	.730**	.357**	.427**	.512**	—				
6. Hinting Task	.468**	.259**	.360**	.363**	.427**	—			
7. PS Composite	−.372**	−.339**	−.295**	−.195*	−.368**	−.404**	—		
8. LogDES	−.321**	−.337**	−.146	−.322**	−.234**	−.229**	.473**	—	
9. log_MDI	−.383**	−.374**	−.276**	−.308**	−.267**	−.216*	.491**	.764**	—

Note. MSCEIT = Mayer-Salovey-Caruso Emotional Intelligence Test; PS Composite = Psychosis Symptom Composite; LogDES = log-transformed Dissociative Experiences Scale; log_MDI = log-transformed Multidimensional Inventory of Dissociation Total. All correlations are Spearman's rho. * $p < 0.05$. ** $p < 0.01$, two-tailed.
